# Innovative Materials Based on Epoxy Resin for Use as Seat Elements in Bulk Transport

**DOI:** 10.3390/ma17081829

**Published:** 2024-04-16

**Authors:** Angelika Plota-Pietrzak, Leszek Czechowski, Sebastian Miszczak, Anna Masek

**Affiliations:** 1 Institute of Polymer and Dye Technology, Faculty of Chemistry, Lodz University of Technology, 90-537 Lodz, Poland; angelika.plota@dokt.p.lodz.pl; 2 Department of Strength of Materials, Lodz University of Technology, 90-537 Lodz, Poland; leszek.czechowski@p.lodz.pl; 3 Institute of Materials Science and Engineering, Lodz University of Technology, 90-537 Lodz, Poland; sebastian.miszczak@p.lodz.pl

**Keywords:** epoxy resin, polylactide, flavonoid, aging tests, rail vehicles, seat elements

## Abstract

The subject of this research is the development of epoxy composites with a defined service life for the purpose of seat elements in rail vehicles, which will be more environmentally friendly. The produced materials based on epoxy resin filled with PLA or PLA and quercetin were subjected to solar aging tests for 800 h to investigate the impact of the additives used on the aging behavior of the epoxy matrix. Firstly, the TGA analysis showed that the use of the proposed additives allowed for the maintenance of the thermal stability of the epoxy resin. Moreover, based on an optical microscopy test, it was noticed that the introduction of PLA and PLA with quercetin did not contribute to an increase in matrix defects. The one-directional tensile tests carried out before and after solar aging showed that the presence of polylactide in epoxy composites causes a slight growth of the stiffness and strength. Based on contact angle and color change measurements, it was found that quercetin was oxidized, thus ensuring protection for the epoxy matrix. This phenomenon was confirmed by FTIR study, where the carbonyl index (CI) value for the R-PLA-Q composite was lower than for the reference sample. The obtained composite structures may be a good alternative to traditionally used systems as seat elements in rail vehicles, which are not only characterized by high aging resistance but are also more eco-friendly.

## 1. Introduction

The intensive development of science and various technologies resulting from the constantly growing demand for innovative materials as well as the growing human awareness and legal changes related to the replacement of common raw materials with those that come from renewable sources are currently the main factors of progress in modern science [1,2,3,4,5,6,7,8,9,10].

Petroleum-based epoxy resin is a thermosetting polymer that is most commonly used in composite materials [11]. Due to high mechanical strength, good adhesiveness, low cost, high thermal stability, good chemical resistance, and ease of use of epoxy resins, they are successfully used in load-bearing applications (aerospace, automotive, construction, and marine). In addition, they are also utilized in the production of coatings, adhesives, insulation, and high-performance composites [4,12,13,14,15,16]. However, due to the insolubility and non-fusibility of epoxy resins, they are difficult to recycle, which results in a waste of resources and limits their applications [17]. This problem was solved in 2011 by Ludwik Leibler and co-workers [18], who discovered a new class of materials, namely epoxy vitrimers, which are reversible covalently cross-linked networks and, in addition to having excellent mechanical behavior, are characterized by good reprocessability and recyclability [19,20,21]. An interesting approach to obtaining bio-based epoxy vitrimers was demonstrated by Liu et al. (2021) [22], who synthesized two dynamic imine bond curing agents using m-xylylenediamine, 1,6-hexanediamine, and biomass energy vanillin as raw materials, which were then cured with DGEBA. The authors observed that the functional properties of these composites were comparable or even better than those for conventional epoxy resin; however, they exhibited degradable characteristics due to the hydrolysis of imine bonds. Another disadvantage of cured epoxy materials is that they can be stiff and brittle, depending on the hardener used [23]. Therefore, scientists are constantly working on methods related to improving their physical performance [24].

One of the common ways to refine their physical properties is the addition of fillers to the epoxy matrix, which not only reduce the production costs but also improve the mechanical properties of these composites [25,26]. However, in many cases, an additional functionalization of the filler is necessary to improve the interphase between the polymer matrix and the additive and, consequently, the overall composite properties. There are many examples in the available literature in which fillers of natural (jute flour, coconut shell, waste peanut shell, banana bark, etc.) or synthetic origin (aramid and graphite fibers, etc.) were used for the epoxy resin [8,27]. Natural fibers are lighter, cheaper, and better in terms of natural environment protection [28]. For example, Salasinska et al. [29] prepared the epoxy composites modified with ground walnut shell in the amounts of 20, 30, 40 and 50 wt%. It was found that the incorporation of these organic waste fillers improved the thermal stability, stiffness, and hardness of epoxy composites; nonetheless, their tensile strength and impact resistance decreased in comparison to the unmodified resin. Similarly, Barczewski et al. (2019) [30] observed a reduction in the mechanical performance of epoxy materials filled with sunflower husk. Another example of the use of ecological fillers in the form of hemp fibers for epoxy resin was presented by Gargol et al. (2021) [31], whose test results showed that 30 wt% of these fibers causes a decrease in strength from 40.2 MPa to 4.2 MPa compared to the unfilled sample. In addition, the hardness of these composites also decreased. Therefore, currently, the most commonly used fibers in the epoxy matrix are glass and carbon fibers in the form of mats or short-cut fibers due to their outstanding mechanical behavior [27].

Another important aspect is the service life of epoxy resin products. During operation, these materials may be exposed to various environmental factors (solar radiation, elevated temperature, and many others) [32]. For example, epoxy resin composites are commonly used in the aerospace industry where they are exposed to high-energy gamma radiation [33]. Therefore, it is important to design the products so that they are safe and non-defective throughout their lifetime. For this purpose, simulated aging tests are usually carried out in appropriate chambers [34]. Moreover, due to the flammability of epoxy resin-based materials and the high fire hazard, appropriate chemicals are required to ensure their safe use and to provide them with flame-retardant properties. For example, Wang et al. (2021) [35] prepared an effective fire retardant system for epoxy resin by introducing 15 wt.% silica and a 5 wt.% phenethyl-bridged 9,10-dihydro-9-oxa-10-phosphaphenanthrene-10-oxide (DOPO) derivative. The limiting oxygen index increased from 21.8 to 30.2% compared to the pure epoxy resin sample.

Exposure to solar radiation and elevated temperature causes photochemical damage, which results in the degradation of composite elements and deterioration of their performance properties. In order to prevent the undesirable changes in polymer products caused by aging processes, there are many ways to improve their aging resistance [36]. Currently, the most common method to mitigate the photooxidation effects is to introduce antioxidant compounds or a small amount of inorganic fillers to the polymer matrix [27,37,38,39]. Antioxidants are active compounds that are able to reduce the rate of photooxidation by removing or inactivating free radicals through donating hydrogen atoms, which in turn interrupts the chain reactions [40]. However, some researchers observed that these substances added to the polymer matrix cause its relaxation or affect the glass transition temperature (Tg) [41]. Siddiqui et al. (2021) [42] demonstrated that thymol, carvacrol, limonene, and cinnamaldehyde introduced into the polylactide (PLA) matrix resulted in a decrease in the Tg value due to the plasticizing effect of these compounds. On the other hand, in the case of the second group of additives, i.e., inorganic fillers, Khotbehsara et al. (2020) [43] showed that the incorporation of hydrated alumina powder and fly ash can significantly improve the UV resistance of epoxy resin. In another study, Wang et al. (2023) [44] explored the role of graphene in enhancing the resistance to radiation of epoxy resin. Their results showed that as a result of gamma irradiation, the glass transition temperature, nano-indentation depth, and hardness of graphene-modified epoxy resin composites decreased by 20.32%, 416.3 nm, and 16.00%, respectively, whereas for unmodified epoxy resin, they decreased by 30.34%, 502.1 nm, and 41.82%, respectively. It was proved that the addition of graphene nanoparticles can reduce the free radical content in epoxy resin and inhibit its aging process. According to Zhang et al. (2015) [45], this phenomenon can be related to the adsorption of oxygen-containing free radicals by the surface defects of graphene oxide, which are then consumed by surface oxygen-containing groups (e.g., quinones) via single-electron reduction, ultimately quenching oxygen.

Taking into account all the aspects described above, the aim of this work was to design composites based on epoxy resin which, as a result of appropriately selected additives of natural origin, in addition to increasing their recycling potential and being more environmentally friendly, will also retain their functional properties (e.g., durability, hardness, and thermal resistance) and will be characterized by improved resistance to various environmental factors during their operation. Therefore, polylactide in the powder form has been proposed as a biofiller, and natural quercetin was used to provide the epoxy matrix an antioxidant effect. The material compositions proposed in this article may be an excellent alternative to solutions commonly used in the epoxy materials industry, which additionally solve the problems related to the use of compounds of natural origin in epoxy composites described by other authors. Moreover, such an approach has not been described in the literature so far; therefore, it is definitely a novelty in science.

## 2. Materials and Methods

### 2.1. Materials

An epoxy resin containing flame retardants (ASSET 1011) supplied by New Era Materials (Modlniczka, Poland) was used as a polymer matrix. According to the MSDS provided by the resin producer, this mixture contained about 73 wt.% pure epoxy resin, less than 18 wt.% poly(ammonium phosphate), and about 9 wt.% expanded graphite. The second polymer used in this study was an eco-friendly polylactide in the powder form (PLA RXP 7501, MFI = 75 g/10 min) obtained from Resinex (Warsaw, Poland). Moreover, natural quercetin (quercetin hydrate, ≥95%) supplied by Sigma-Aldrich (Steinheim, Germany) was incorporated into the epoxy resin containing polylactide. Fiberglass fabric (RXT 350 g/cm^2^) obtained from Rymatex (Rymanów, Poland) was used as a reinforcement in the produced composites.

### 2.2. Preparation of Composites

The process of producing composites consisted of 3 stages: preparation of the prepreg and its plasticization, cross-linking, and hardening. First, all ingredients were weighed on a laboratory balance by sieving them through a strainer directly into a beaker (resin) or a plastic cup (polylactide and quercetin). Then, the additives were added to the resin through a strainer and homogenized for about 3 min. The weight composition of the prepared samples is shown in Table 1. The next step was the prepreg preparation. Each prepreg consisted of 3 layers of epoxy resin with or without additives and 3 layers of fiberglass fabric. Ready prepregs were processed (plasticization, cross-linking, and hardening) in accordance with the conditions presented in Table 2.

### 2.3. Accelerated Aging Test

Fabricated composites were exposed to the accelerated aging test in an Atlas SC 340 MHG solar simulator climate chamber from AMETEK Inc. (Berwyn, IL, USA) that was equipped with a 2500 W MHG lamp. A unique range of solar radiation (UV, Vis, and IR) was provided by a rare-earth halogen lamp. The samples were aged for 800 h at a temperature of 70 °C with maximum solar radiation throughout the aging process.

### 2.4. Surface Wettability and Determination of Surface Energy

Surface energy is a measure of the interaction of a solid substrate with the liquid that wets the tested material, and its determination consists in determining the contact angles by appropriately selected liquids, the surface tensions of which are known, including their dispersion and polar parts. In this case, distilled water, diiodomethane, and ethylene glycol were used. In addition, the Owens–Wendt–Rabel–Kealble (OWRK) model was applied, thanks to which the surface energy was calculated including its polar and dispersion components:

(1) $$E=E_{P}+E_{D}$$

(2) $$\frac{\sigma_{L}\left(1+cos\Theta \right)}{2\sqrt{\sigma_{L}^{D}}}=\sqrt{\sigma_{S}^{P}}\cdot \sqrt{\frac{\sigma_{L}^{P}}{\sigma_{L}^{D}}}+\sqrt{\sigma_{S}^{D}}\;as\;a\;linear\;function\;Y\;=\;\mathrm{aX}\;+\;b$$

(3) $$Y=\frac{\gamma_{L}\left(1+cos\Theta \right)}{2\sqrt{\sigma_{L}^{D}}},X=\sqrt{\frac{\sigma_{L}^{P}}{\sigma_{L}^{D}}},a=\sqrt{\sigma_{S}^{P}},b=\sqrt{\sigma_{S}^{D}}$$

(4) $$E_{P}=a^{2}=\sigma_{S}^{P}\;oraz\;E_{D}=b^{2}=\sigma_{S}^{D}$$

The ratio of polar and dispersion constituents affects the wetting and adhesion of the two tested phases. The polar component of surface energy is the sum of acid–base, hydrogen, and inductive forces, while the dispersion part is the magnitude of intermolecular forces such as Van der Waals forces. The more similar the ratio of these two components, the greater the interactions and the stronger the adhesion between both phases [46,47].

The test was carried out using a goniometer (OCA 15EC) equipped with a camera from DataPhysics Instruments GmbH (Filderstadt, Germany), a Braun DS-D 1000 SF syringe, and SCA20 software and it consisted of placing 6 drops of each measuring liquid with a volume of 1 µL on the substrate of each test sample from the side where the last layer was the resin. The obtained results from the static contact angles were used to determine the surface energy of the tested materials by the SCA20 program (version 1.0).

### 2.5. FTIR Spectroscopy

Fourier transform infrared spectroscopy (FTIR) was performed using a Nicolet 6700 FT-IR spectrometer from Thermo Scientific (Waltham, MA, USA), which was equipped with a diamond Smart Orbit ATR sampling equipment. The FTIR spectra were recorded in the range of 4000–400 cm^−1^ using 64 scans and at a resolution of 4 cm^−1^. The test was performed for all tested materials before and after 800 h of solar aging. On the basis of the spectra obtained, the carbonyl index was calculated, which can be the basis for the assessment of the material degradation progress [48,49,50]:

(5) $$CI=\frac{I_{C=O}}{I_{C-H}}$$

### 2.6. Color Change Measurements

The color change analysis of samples based on the epoxy resin was carried out according to the PN-EN ISO 105-J01 standard by applying the UV-VIS CM-3600d spectrometer from Konica Minolta Sensing, Inc. (Osaka, Japan). Each sample was tested in at least three different places. Then, the obtained results were interpreted in the CIE-Lab space, thanks to which a color description was obtained by designating three coordinates (L*, a*, and b*). The first of them is L—a lightness indicator with a value range from 0 to 100, where 0 means black and 100 means diffuse white. The second coordinate is the a* parameter, whose positive value means red and negative value means green. For the third parameter (b*), a positive value means yellow, and a negative one means blue [51,52]. The color change of the tested materials was calculated with the formula below [53]:

(6) $$\Delta E=\sqrt{{\left(\Delta a\right)}^{2}+{\left(\Delta b\right)}^{2}+{\left(\Delta L\right)}^{2}}$$

### 2.7. Hardness Tests

Hardness measurements were conducted according to the PN-71/C-04238 standard. Two digital hardness meters were used, one on the Shore “C” scale and one on the Shore “D” scale (Zwick GmbH&Co, Ulm, Germany, pressure 50 N). Hardness tests were performed for all samples before and after 800 h of aging.

### 2.8. Optical Microscopy

Images of microstructures obtained on cross-sections were made using a Keyence VHX-950F microscope equipped with VH-Z100R lens. Hybrid lighting mode (bright and dark field) at 200× magnification was used.

### 2.9. Thermogravimetric Analysis

Thermogravimetric analysis (TGA) allowed for the assessment of the influence of applied additives incorporated into the epoxy resin on its thermal degradation process, which involves the mass change of a material as a function of raising temperature. During the analysis, a Mettler Toledo TGA/DSC 1 STARe device equipped with a GC10 gas controller (Greifensee, Switzerland) was used. The measurement was carried out in the oxidizing atmosphere of synthetic air in the temperature range of 25–1000 °C, at a heating rate of 20 °C/min, and an air flow of 60 cm^3^/min. The test samples were placed in crucibles made of alumina.

### 2.10. One-Directional Tensile Test

**Figure 1:**
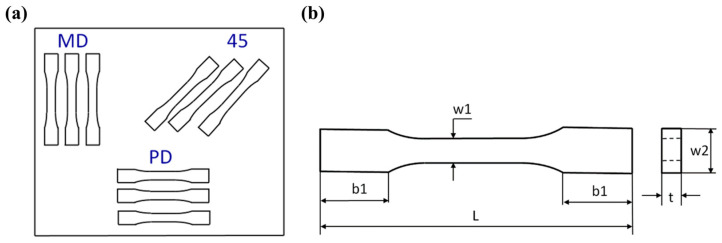
Scheme of taken samples (**a**) and drawing of sample (**b**).

The tensile tests were carried out on the basis of standard UNE EN ISO 527-1:2020-01 using an INSTRON testing machine. The constant speed of the moveable traverse was assumed to be stable and equal to 2 mm/min. As a result of tests, the diagrams of force vs. elongation were obtained. The samples were cut out from the plate according to the scheme shown in Figure 1a, where the symbols mean the following: MD—main direction; PD—perpendicular direction; and 45—angle orientated to the plate edges. The dimensions of the samples were based on the aforementioned standard: L = 170 mm, b1 = 40 mm, w1 = 10 mm, w2 = 20 mm, and mean thickness t = 3 mm (Figure 1b). The Young’s modulus of the composite was determined by using an extensometer with a gauge length of 50 mm.

## 3. Results and Discussion

### 3.1. Thermogravimetric Analysis (TGA)

**Figure 2:**
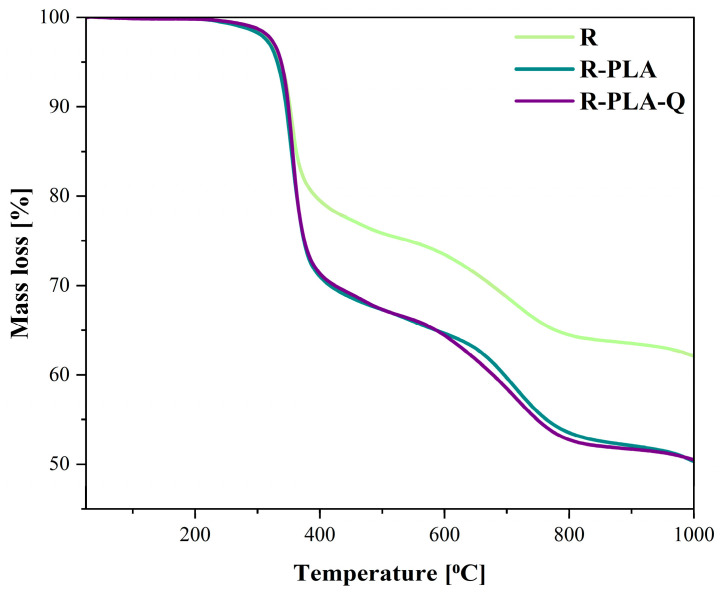
Thermal decomposition analysis of the tested materials recorded in the temperature range of 25–1000 °C.

Thermogravimetric analysis was carried out to assess the thermal stability of epoxy resin samples with natural additives (Figure 2 and Table 3). Table 3 summarizes the obtained values of T_5%_, T_10%_, T_20%_, T_30%_, and the residues for pure epoxy resin (R), epoxy resin containing polylactide (R-PLA), and epoxy resin with polylactide and quercetin (R-PLA-Q) before solar aging, which refer to mass changes of 5%, 10%, 20%, and 30%, respectively.

The thermal decomposition of all tested samples occurred in two step degradation. The first step occurred approximately between 230 and 400 °C followed by the second step around 400 to 780 °C. Analyzing the T_5%_ temperature values in Table 3, which are commonly considered as the initial temperature of the thermal decomposition of the samples, it can be observed that the addition of polylactide only minimally reduced the thermal resistance of the epoxy resin, while the addition of both polylactide and quercetin allowed for the preservation of this property. Much greater differences are more visible in the further stage of material degradation, when the temperature at which the mass change is 30% is equal to 676 °C for the reference resin sample and 414 °C for the composite with polylactide addition. It should be emphasized, however, that the content of quercetin in the R-PLA composite improves the thermal resistance of the material at various stages of degradation. Moreover, the weight fraction showing the percentage of unburned residue of the sample after the measurement is probably due to the presence of the glass fabric as well as the presence of mineral additives in the resin mixture (e.g., flame retardants, etc.).

### 3.2. Optical Microscopy

**Figure 3:**
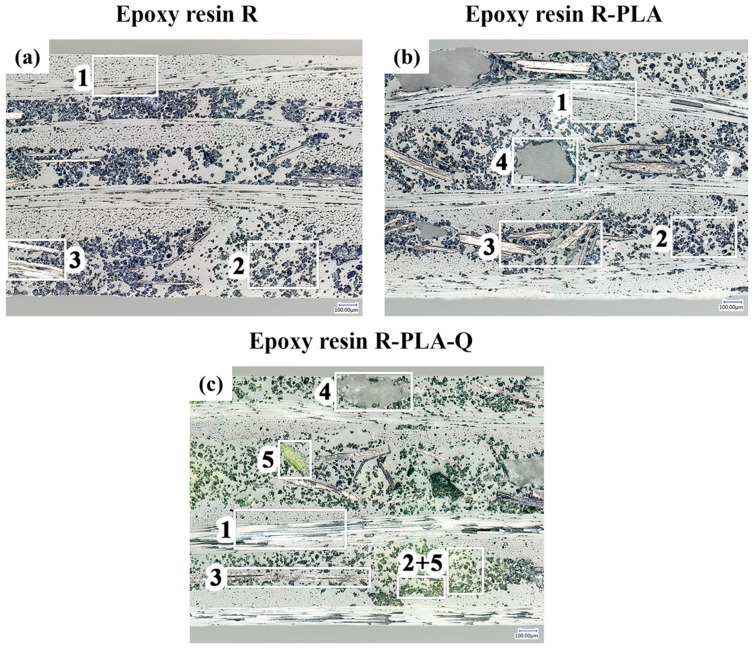
Cross-section images of (**a**) pure epoxy resin composite (R), (**b**) epoxy resin composite containing polylactide (R-PLA), and (**c**) epoxy resin composite with polylactide and quercetin (R-PLA-Q). 1—glass fabric layers; 2—epoxy matrix with APP particles; 3—graphite flakes; 4—PLA particles; 5—quercetin particles. Microstructures obtained at magnification of 200×.

In order to assess the internal microstructure of the composites, which may affect their aging process, observations were carried out using optical microscopy. The internal microstructures of the composites were examined in cross-sections. Microscopic photos taken at the magnification of 200× are shown in Figure 3.

The cross-section of composite R, shown in Figure 3a, reveals its microstructure consisting of three layers of glass fabric (1) and an epoxy matrix containing dispersed flame-retardant additives: ammonium polyphosphate (2) and graphite (3). The share of the APP as a main flame-retardant additive, determined on the basis of calculating the area occupied by it, amounted to approx. 17%, which is close to the manufacturer’s declaration. Disturbances in the dispersion of additives in the form of areas of high and low density are visible. Figure 3b shows the cross-section microstructure of the R-PLA composite with the addition of polylactide. The PLA is visible in the form of particles of a separate phase (4) with small fragmentation and large size. The size and distribution of PLA particles may be the result of aggregation and its secondary crystallization during the composite hardening stage. In the case of large-sized crystallites, they may disturb the arrangement of glass fiber laminate layers, which can be observed in the upper part of the microstructure. Figure 3c shows the cross-section microstructure of the R-PLA-Q composite with the addition of polylactide and quercetin. The appearance of the microstructure is similar to the R-PLA composite with the addition of polylactide and differs only in the presence of yellow-colored areas containing quercetin particles (5).

The introduction of both additives did not contribute to an increase in the degree of matrix defectivity, e.g., in the form of porosity. This indicates good integration of the introduced additives with the composite matrix. The small size of quercetin particles and the low glass transition temperature of PLA (thanks to which it remained in a “rubbery state” during all three stages of composite processing) could have had a beneficial effect, especially during the plasticization of prepregs, without hindering the plasticization process of the epoxy matrix, which has a key impact on the formation of porosity and, as a consequence, on mechanical strength.

### 3.3. Surface Wettability and Determination of Surface Energy

**Figure 4:**
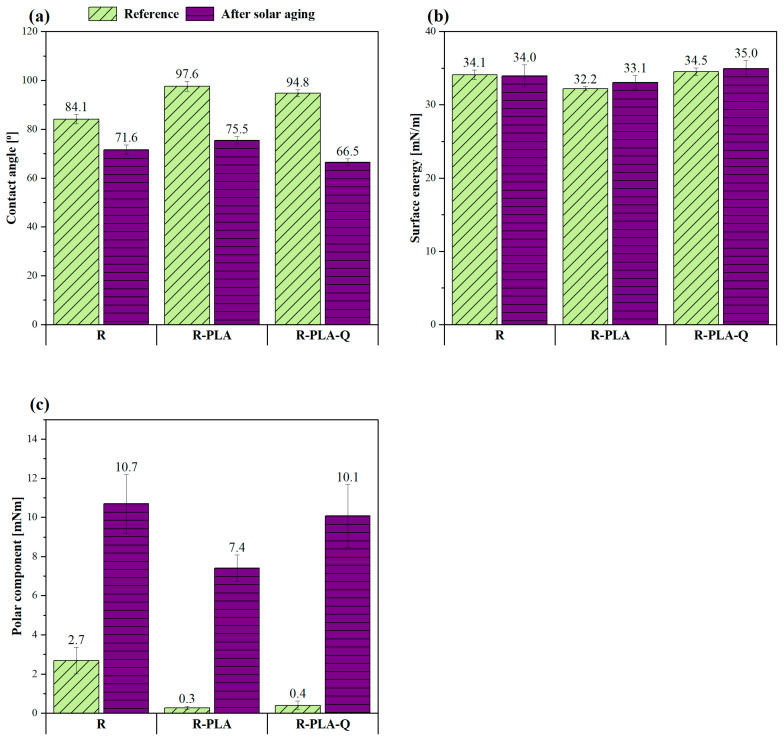
Changes in water contact angle values (**a**), surface energy (**b**), and its polar component (**c**) caused by 800 h of solar aging. R—epoxy resin; PLA—polylactide; Q—quercetin.

To better understand the effect of the accelerated aging test and the presence of applied natural additives on the surface properties of epoxy resin, contact angle measurements were carried out and the surface energy was calculated by applying the Owens–Wendt–Rabel–Kealble approach (Figure 4). This method consists in determining the contact angles by using polar and non-polar fluids. In this case, distilled water, diiodomethane, and ethylene glycol were used as measuring liquids. Overall, wettability is a very important material parameter that can be useful in assessing the surface stabilization of a modified polymer product. Comparing the average values of the water contact angles before aging in Figure 4a, it can be seen that the addition of both PLA itself and PLA with quercetin caused an increase in these values compared to the pure resin sample and thus changed the nature of these surfaces to be more hydrophobic, which showed worse wettability. On the other hand, all samples after solar aging exhibited a more hydrophilic character as they were less than 90°. This proves their good surface wettability but also lower resistance to oxidation processes. The lowest value of the water contact angle was observed for the sample containing quercetin, which may indicate the oxidation reactions occurring in the structure of this polyphenol, which are caused by the aging process [54,55]. Moreover, when analyzing the values of surface energy before and after 800 h of solar aging in Figure 4b, it can be observed that they did not change significantly, but much larger differences could be seen in the values of its polar component (Figure 4c). In the case of the R-PLA-Q composite, the difference in the polar constituent before and after the aging process was 9.7 mN/m, which proves its greatest susceptibility to oxidation processes and it is consistent with the reported water contact angle measurements [56,57]. On the other hand, the pure epoxy resin and that with only polylactide content showed similar surface properties, and in their case, the difference in the polar component before and after solar aging was about 7 mN/m.

### 3.4. FTIR Spectroscopy

**Figure 5:**
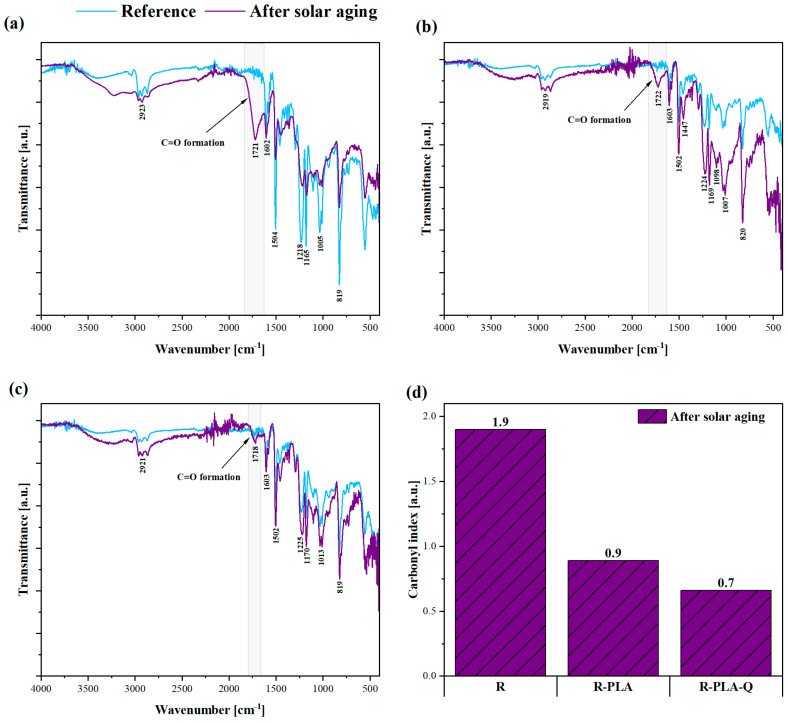
FTIR spectra of (**a**) pure epoxy resin; (**b**) epoxy resin—PLA; (**c**) epoxy resin—PLA—quercetin, before and after 800 h of solar aging; and (**d**) carbonyl index values determined for all samples after aging.

FTIR analysis was performed to examine the degree of degradation of the tested polymer materials, which is possible by analyzing the differences in the intensity of the bands indicating the presence of CH bonds or by the appearance of new bands that indicate the presence of oxygen connections, such as C=O, C–O, O–H, O–C=O, and C=C. Figure 5a–c show the obtained FTIR spectra for the tested samples, on the basis of which the values of the carbonyl index were calculated (Figure 5d). In general, the carbonyl index informs about the progress of oxidation and decomposition of polymeric materials. By analyzing the FTIR spectra before and after the aging process, one can observe changes in the intensity of the peaks at a wavenumber of about 2920 cm^−1^ (asymmetric vibrations stretching the methylene group CH_2_) and the newly appearing band at about 1720 cm^−1^, which indicates the presence of C=O bonds in the examined structures, which decompose to carbon dioxide and water during oxidation. Similarly, Wu et al. (2022) [58] observed the formation of a new absorption peak at a wavenumber of 1730 cm^−1^ corresponding to the stretching vibration of the C=O group. According to the authors, this chromophore can be responsible for the yellowing of epoxy resin during aging. Taking into account the calculated values of carbonyl indexes, it is visible that the R-PLA-Q sample was characterized by the highest resistance to oxidation processes (CI = 0.7). This is due to the fact that during solar aging, the oxidation reactions occurred in the polyphenol itself, which thus provided an effective protection for the polymer matrix. Moreover, it was observed that both the polylactide itself and the polylactide with the addition of quercetin incorporated into the epoxy resin acted effectively and delayed the polymer matrix degradation process. Table 4 presents all transmission bands assigned to the chemical groups (bonds) that changed as a result of solar aging.

### 3.5. Color Change Measurements

**Figure 6:**
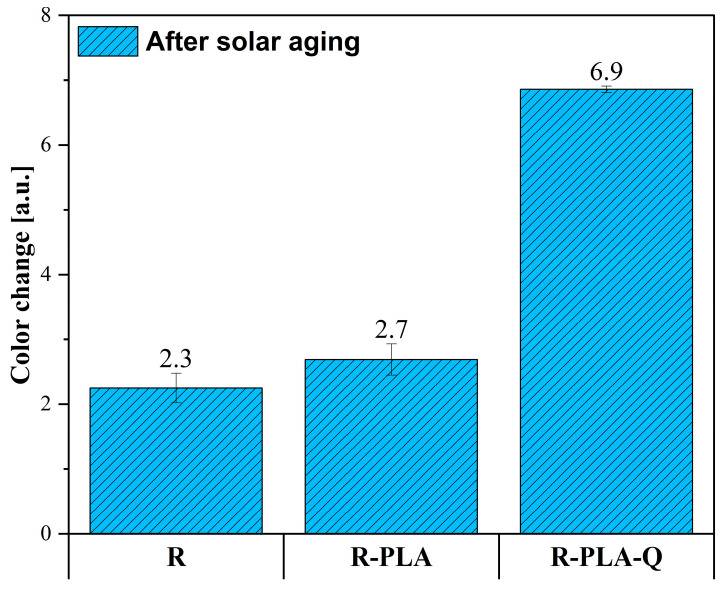
Color change (ΔE) of tested materials caused by 800 h of solar aging.

Based on the spectrophotometric measurements, three color parameters were determined: L*, a*, and b*. The obtained values are presented in Table 5, on the basis of which the color change (ΔE) presented in Figure 6 was calculated.

When analyzing changes in the lightness index (L*) values before and after aging, no significant changes were noted in the case of the reference resin sample and with the addition of polylactide, while for the R-PLA-Q material, the value of this parameter decreased as a result of solar aging. This means that it has become much darker. Additionally, for all samples, there was an increase in the value of the a* parameter, denoting a color shift of these materials towards shades of red. Analyzing the values of the b* parameter before and after the aging process, it is visible that in the case of the reference resin sample and with the addition of polylactide and quercetin, these values decreased, which means a color change more towards shades of blue, while for the R-PLA composite, there was a change towards shades of green. The greatest color change (6.9), which was easily noticeable for a potential observer, was obtained for the R-PLA-Q material, which could be directly related to the oxidation reactions occurring in this polyphenol. According to the available literature, flavonoids such as quercetin exhibit strong antioxidant properties due to the presence of numerous double bonds and hydroxyl groups in their structure, thanks to which they can act as scavengers of free radicals, reacting with them under the influence of various atmospheric factors, including UVA and UVB radiation [65]. At the same time, an oxidation reaction takes place in the structure of these compounds, which changes their coloration. Such behavior of quercetin allows for longer preservation of the properties of the polymer matrix and thus the use of the polymer product.

### 3.6. Hardness Tests

**Figure 7:**
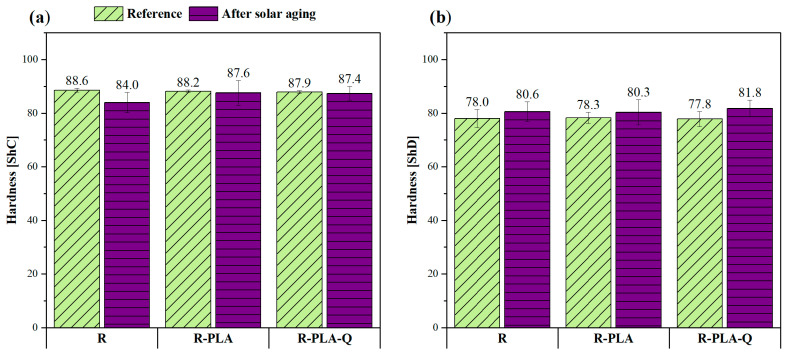
Results of hardness tests on the Shore “C” scale (**a**) and on the Shore “D” scale (**b**) before and after solar aging.

The hardness test of the epoxy resin composites was performed at six different places, both from the surface, where the last layer was resin, and the fiberglass fabric. The obtained average hardness values on the Shore “C” and “D” scales are presented in Figure 7. The addition of polylactide and polylactide with quercetin had no effect on the reported values of this parameter before the aging process: all samples showed a Shore “C” hardness of about 88 ShC and a Shore “D” hardness of about 78 ShD. The same tendency was observed after 800 h of solar aging, because the changes caused by this process were practically imperceptible in terms of the hardness of these composites. This is a good sign that the biofiller in the form of PLA powder does not deteriorate the mechanical properties of the epoxy resin, while in the available literature, most of the natural fillers had a negative impact on this parameter. For example, in studies performed by Gargol et al. (2021) [31], the hardness of epoxy resin composites filled with 30 wt% hemp fibers decreased from 84 ShD to 73 ShD. Therefore, it can be concluded that the samples produced were characterized by good compatibility and no structural defects. Moreover, if the Shore “D” hardness is greater than 60 ShD, the test material is very hard. In this case, all the samples, both before and after aging, had a hardness greater than 60 ShD, which means that they were very hard and, therefore, they would be perfect for areas where such durability is necessary.

### 3.7. One-Directional Tensile Test

**Figure 8:**
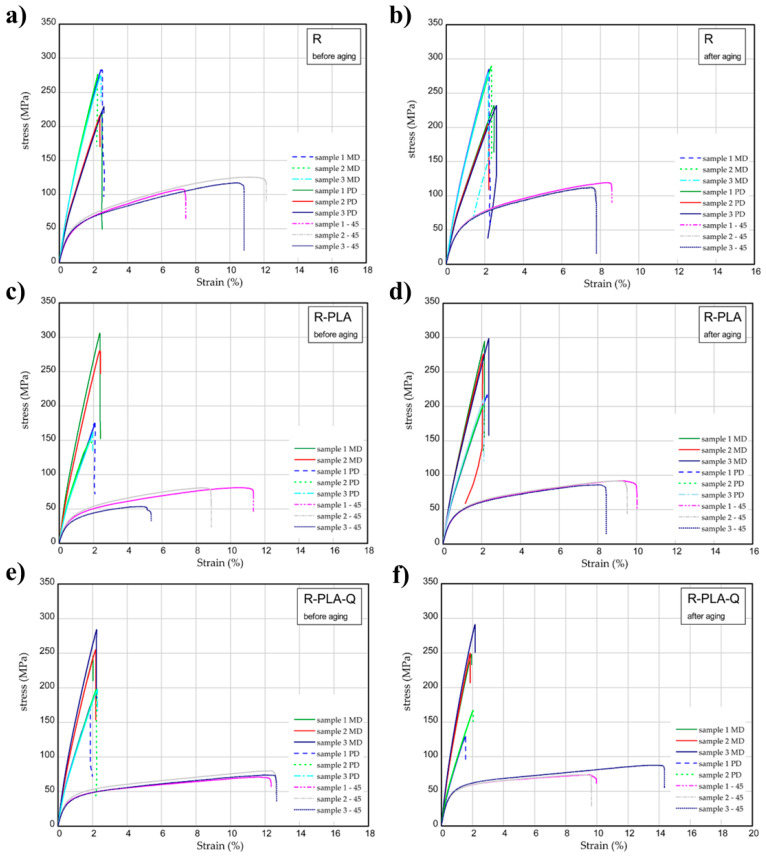
Curves of tension for pure epoxy resin (R) (**a**,**b**), R-PLA (**c**,**d**) and R-PLA-Q (**e**,**f**) before aging and after solar aging tests.

The results of tensile tests of samples examined before and after the aging process are illustrated in Figure 8. The mechanical parameters were determined for three directions (MD, PD, and 45). Figure 8a,b present the strain expressed in % vs. normal stress in MPa for the reference sample. The maximum stress and Young’s modulus for MD samples were 277–283 MPa and 17.3–18.3 GPa, respectively. In the case of PD samples, these values obtained were lower by 15–20% in comparison to MD samples. Taking a look at the 45 samples, the mechanical parameters are lower (usually about two times). After the solar aging process, the strength of composite seems to be higher (Figure 8b). Characteristic parameters of analyzed samples were inserted in Table 6. Analyzing the next charts (Figure 8c–f), in general, the presence of PLA in samples causes a slight growth in the strength and stiffness, but the addition of 2 wt.% of quercetin can lower the mechanical parameters even after solar radiation.

## 4. Conclusions

The following paper proposes the use of a biofiller in the form of polylactide and quercetin for epoxy resin composites intended for seat elements in bulk transport. Their processing was carried out in three stages: prepreg preparation and plasticization, cross-linking, and hardening. The aim of this research was to investigate the effect of the selected additives on the aging behavior of the epoxy matrix. Therefore, the produced samples were subjected to solar aging at a temperature of 70 °C for 800 h.

Microstructure analysis of these composites showed that the introduction of both additives did not contribute to an increase in the matrix defectivity, e.g., in the form of porosity. The proper integration of additives into the composite matrix and low porosity could have a beneficial effect on good mechanical properties, which were significantly better compared to other bioadditives described in the literature. Moreover, based on the TGA results, it was observed that the incorporation of PLA and quercetin allowed for the maintenance of the thermal resistance of the epoxy resin. In the case of one-directional tensile tests, a slight growth in the strength and stiffness was noted for the composite containing polylactide.

Based on the contact angle and color change measurements, a high susceptibility of quercetin to oxidizing was observed; however, it is possible that by its oxidation and reaction with free radicals, the polymer matrix was protected during exposure to elevated temperature and solar radiation. This was confirmed by the FTIR analysis, where the value of the CI for the composite with the addition of polylactide and quercetin was lower than for the reference sample. In addition, the hardness of the tested composites did not deteriorate as a result of aging tests. Therefore, it can be concluded that the samples produced were characterized by good compatibility and no structural defects. The obtained composite structures may be a good alternative to traditionally used systems as seat elements in rail vehicles, which are not only characterized by high aging resistance but are also more eco-friendly.

## Figures and Tables

**Table 1:** Weight composition of samples based on epoxy resin.

Mixture	Weight Composition		
	Epoxy Resin [phr]	Polylactide [phr]	Quercetin [phr]
1	100	-	-
2	100	15	-
3	100	15	2

**Table 2:** Processing conditions of epoxy resin-based composites.

Processing Conditions							
STAGE 1 (Prepreg Plasticization)			STAGE 2 (Cross-Linking)			STAGE 3 (Hardening)	
T [°C]	t [min]	p [bar]	T [°C]	t [min]	p [bar]	T [°C]	t [min]
90	5	6	130	8	10	136	45

**Table 3:** Temperatures of the mass loss observed for the tested materials, where T_x%_ is a temperature at which the mass change is x% (5, 10, 20, and 30, respectively). R—epoxy resin; PLA—polylactide; Q—quercetin.

Sample	Temperatures of the Mass Change [°C]				
	T_5%_	T_10%_	T_20%_	T_30%_	Residue [%]
R	337	350	390	676	62.1
R-PLA	332	345	361	414	50.4
R-PLA-Q	337	348	361	422	50.5

**Table 4:** Transmission bands assigned to the chemical groups (bonds) that changed due to aging in the tested materials.

Wavenumber (cm^−1^)	Assignment	Ref.
~3300	OH stretching	[59]
2923	CH_2_ stretching (asymmetric)	[60]
2868	CH_2_ stretching (symmetric)	[60]
1721	C=O stretching	[58]
1603	C=C aromatic ring stretching	[61]
1502	C-H aromatic	[62]
1447	CH_2_ deformation	[63]
1218	Stretching of epoxide -C-O bonds	[61]
1165	C-C stretching	[64]
1005	C-O stretching	[62]
819	C-O-C of terminal oxirane group of epoxy system	[61]

**Table 5:** L*, a*, and b* coordinates determined in the CIE-Lab space for all tested samples before and after solar aging. R—epoxy resin; PLA—polylactide; Q—quercetin.

Sample	Before Aging			After Aging		
	L*	a*	b*	L*	a*	b*
R	35.0	−0.6	3.7	35.3	0.2	2.6
R-PLA	35.3	−0.3	2.5	36.0	0.7	4.7
R-PLA-Q	35.6	−4.4	8.1	32.9	−0.2	3.5

**Table 6:** Main values of Young’ modulus in GPa and maximum stress in MPa.

Young’s Modulus (GPa)						
Sample	Before Aging			After Aging		
	MD	PD	45	MD	PD	45
R	17.31 ± 0.20	14.36 ± 0.40	9.34 ± 0.65	18.43 ± 0.24	14.78 ± 0.45	10.39 ± 0.18
R-PLA	17.95 ± 2.14	12.91 ± 0.28	7.28 ± 0.49	18.41 ± 0.32	15.63 ± 0.41	9.51 ± 0.18
R-PLA-Q	18.03 ± 1.12	14.51 ± 0.37	8.28 ± 0.41	19.10 ± 0.86	13.21 ± 0.58	10.55 ± 0.25
**Maximum Stress (MPa)**						
**Sample**	**Before Aging**			**After Aging**		
	**MD**	**PD**	**45**	**MD**	**PD**	**45**
R	277.7 ± 4.6	223.6 ± 6.7	116.9 ± 8.9	284.9 ± 4.7	223.8 ± 14.2	114.9 ± 4.0
R-PLA	293.3 ± 18.2	162.4 ± 11.8	72.0 ± 15.8	289.9 ± 12.2	210.3 ± 8.4	90.0 ± 3.4
R-PLA-Q	261.7 ± 19.9	189.8 ± 14.0	75.1 ± 4.6	262.8 ± 24.3	147.9 ± 26.7	78.6 ± 7.9

## Data Availability

Data are contained within the article.
